# A mechanistic interpretation of relativistic rigid body rotation

**DOI:** 10.1038/s41598-023-35897-9

**Published:** 2023-06-03

**Authors:** Stefan Catheline

**Affiliations:** LabTAU, INSERM, Centre Léon Bérard, Université Lyon 1, Univ Lyon, 69003 Lyon, France

**Keywords:** General relativity and gravity, Astrophysical disks

## Abstract

The starting point of this manuscript is classical rigid body rotation. As it is well known, it contradicts basis of relativity since infinite speed is reached at infinite distance from the rotation center O. In order to fix this problem, a phenomenological circle-based construction using Euclidian trigonometry is first described: the relativistic rigid body rotation. The physical Eulerian acceleration implied by this geometrical construction then sketches future links with Maxwell’s equation and Lense-Thirring effect. More importantly, relativistic rigid body rotation is shown to be compatible with Lorentz transformation and brings new geometrical interpretations of time and space intervals.

## Introduction

The departure point of the manuscript is the relativist description of a physical phenomenon that, like a sea serpent, repeatedly returned several times to question physicists: rigid body rotation. It was indeed omnipresent right at the beginning of modern sciences through, for example, vortex theories of René Descartes or William Kelvin. It was proposed to Albert Einstein by Kurt Gödel as a universe model. It was also the main support used by Lev Landau to introduce general relativity. However, rigid body rotation presents a big flaw of connection: at large enough distances, the speed can reach and overcome light speed. This difficulty can be avoided, and this is the main idea of the following developments. This forms the basis upon which this whole manuscript is built, through the consideration that among the three fields needed for its description, as the rigid body rotation $\overrightarrow{\upomega }$, the velocity $\overrightarrow{\mathrm{v}}$, and the distance ${\overrightarrow{\mathrm{r}}}_{0}$, this last has a special status: it does not curl. An intuitive phenomenological geometrical construction is proposed. In the first part, a mathematical description based on general Eulerian acceleration describes the underlying physics of the relativistic rigid body rotation. It further draws some possible links toward Maxwell’s equation and Lense-Thirring effect. In the last part, a careful geometrical analysis in relation with Lorentz transformation validates the origin of the name: *relativistic* rigid body rotation.

## Theory

From the Euler relativistic equation to the Maxwell equations

The fields $\overrightarrow{\upomega }$, $\overrightarrow{\mathrm{v}}$ et ${\overrightarrow{\mathrm{r}}}_{0}$ are connected by the classical rigid body rotation:

1 $$ {\vec{\text{v}}} = {\vec{\omega }} \wedge {\vec{\text{r}}}_{0} . $$

The velocity thus curls around the rotation and describes a circle. Applying a vectorial product $\wedge {\overrightarrow{\mathrm{r}}}_{0}$ to the previous equation allows to obtain the reciprocal equation:

2 $$ {\vec{\omega }} = \frac{{{\vec{\text{v}}} \wedge {\vec{\text{r}}}_{0} }}{{{\text{r}}_{0}^{2} }}. $$

**Figure 1:**
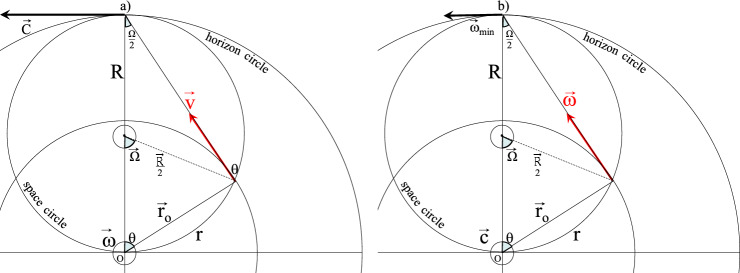
(**a**) Relativistic rigid body rotation and (**b**) relativistic induction, the basis of the rotation theory. Three circles are needed for its construction: the speed circle of radius r_0_, the space circle R/2 and the rotation circle R that defines horizon.

This equation is the mechanical equivalent to Faraday induction in electricity: a velocity field give a vortex similar to “smoke ring”. Such a rotation field follows a closed loop along a circle. As far as the radius is concerned, it always joins these fields along a straight line without ever closing. Replacing this straight segment line ${\overrightarrow{\mathrm{r}}}_{0}$ by a segment circle $\overrightarrow{\mathrm{r}}$, termed a ‘space circle’, is proposed in the rigid body rotation illustrated in Fig. 1. From now on, $\overrightarrow{\upomega }$, $\overrightarrow{\mathrm{v}}$, and $\overrightarrow{\mathrm{r}}$ all follow circles. The asymmetry of these latter fields is fixed by bending space. The space segment circle is thus defined as:

3 $$ {\vec{\text{r}}} = {\vec{\Omega }} \wedge \frac{{{\vec{\text{R}}}}}{2}. $$

The rotation angle $\overrightarrow{\Omega }$ and solid rotation radius $\overrightarrow{\mathrm{R}}$ are defined by construction further in the paragraph. This represents the first postulate of the theory: straight lines do not exist, as physical trajectories are necessarily circular, and thus they obey the rotation: $\overrightarrow{\omega }>\overrightarrow{0}$.

The space circle arbitrarily introduced for symmetry reasons between fields constitute the originality of the rigid body rotation. As it is of finite dimension, the space circle describes a surface in which the rigid body rotation is limited. On its circular boundary at a distance R, a horizon line defines the region of maximum velocity where $\overrightarrow{\upomega }\wedge \overrightarrow{\mathrm{R}}=\overrightarrow{\mathrm{c}}$. An upper bound for velocity that will be called speed of light, is thus inscribed within the very geometrical properties of rigid body rotation.

It tends to classical rigid body rotation $\overrightarrow{\mathrm{r}}\approx {\overrightarrow{\mathrm{r}}}_{0}$ within the limit of Ω << 1; i.e. for weak velocity compared to the speed of light. It can be noted that the space circle size is by construction half of the rotation circle, $\frac{\partial \overrightarrow{\Omega }}{\partial \mathrm{t}}=2\overrightarrow{\upomega }$. It follows that space circle obeys the equation $2\overrightarrow{\upomega }\wedge \frac{\overrightarrow{\mathrm{R}}}{2}=\overrightarrow{\mathrm{c}}$ which can be interpreted as the path taken by any light ray sent from the rotation center. A referential based on the space circle metric r can be qualified as ‘physical’ or ‘real’ whereas the laboratory referential based on the metric r_0_ is ‘mathematical’. Finally, it should be noted that within the manuscript the curvature angle Ω is replaced by its complementary angle $\uptheta =\frac{\uppi }{2}-2\Omega$ for which, by construction, the following relations hold:

4 $$ {\text{cos}} = \frac{{\text{v}}}{{\text{c}}}\;\;{\text{and}}\;\;{\text{sin}} = \sqrt {1 - \frac{{{\text{v}}^{2} }}{{{\text{c}}^{2} }}} . $$

Although James Maxwell had been inspired by analogies with fluid mechanics when he built his theory, William Thomson (Kelvin) blamed the new electromagnetic theory for its lack of common basis with the laws of mechanics stated by Isaac Newton. The reversed point of view of the present theory agrees with Oliver Heaviside, Henri Poincaré, Albert Einstein and many others: Newton’s Law should share the basis of the Maxwell equations. Above all, mechanics does not grant to magnetic fields the central role they deserve. Let us start with the most general expression of acceleration as given by the equation of Leonhard Euler and deduce the expression of electromagnetic force formalized by Hendrik Lorentz. The most general and compact expression of classical acceleration is:

$$ \frac{{{{{\text d}\vec{v}}}}}{{{\text{dt}}}} = \frac{{\partial {\vec{\text{v}}}}}{{\partial {\text{t}}}} + \left( {{{ \vec{v}}}.\overrightarrow {{\text{ grad}}} } \right){{ \vec{v}}}{.} $$

This equation can be developed according to:

5 $$ \frac{{{{{\text d}\vec{v}}}}}{{{\text{dt}}}} = \frac{{\partial {\vec{\text{v}}}}}{{\partial {\text{t}}}} + \frac{{\overrightarrow {{{\text{grad}}}} {\text{v}}^{2} }}{2} + 2{\vec{\omega }} \wedge {{\vec{v},}} $$

With

6 $$ {\vec{\omega }} = \overrightarrow {{{\text{curl}}}} { }\frac{{{\vec{\text{v}}}}}{2}. $$

In rigid body rotation, the following relation holds $\overrightarrow{\mathrm{grad}}\frac{{\mathrm{v}}^{2}}{2}=-\overrightarrow{\upomega }\wedge \overrightarrow{\mathrm{v}}$. Then the expression of acceleration $\overrightarrow{\mathrm{a}}=\frac{\mathrm{d}\overrightarrow{\mathrm{v}}}{\mathrm{dt}}$ becomes:

7 $$ {\vec{\text{a}}} = \frac{{\partial {\vec{\text{v}}}}}{{\partial {\text{t}}}} - \overrightarrow {{{\text{grad}}}} \frac{{{\text{v}}^{2} }}{2}. $$

With Eqs. (6) and (7) embryos of magnetic and electric fields are obtained. However, if in the acceleration of Eq. (7) the gradient expression is very similar to what is expected from an electric field, the curl operator and the related magnetic field is absent. Indeed, going back to Eq. (5), the gradient can be interpreted as a centrifugal acceleration opposed to half of the attractive acceleration of the curl term or in other words, the magnetic acceleration. As a consequence, just by introducing $\overrightarrow{{\mathrm{a}}_{\mathrm{E}}}=-\overrightarrow{\mathrm{grad}}\frac{{\mathrm{v}}^{2}}{2}$ as a definition of the electric acceleration rather than $\overrightarrow{{\mathrm{a}}_{\mathrm{E}}}=+\overrightarrow{\mathrm{grad}}\frac{{\mathrm{v}}^{2}}{2}$, attractive virtue originated from the magnetic acceleration $\overrightarrow{{\mathrm{a}}_{\mathrm{B}}}=2\overrightarrow{\upomega }\wedge \overrightarrow{\mathrm{v}}$ is incorrectly attributed to the electric field. In other words, the electric field changes its sign by including the magnetic acceleration at the origin of the attractive acceleration. The mechanics approach thus fails to account simultaneously for the electric and magnetic fields.

**Figure 2:**
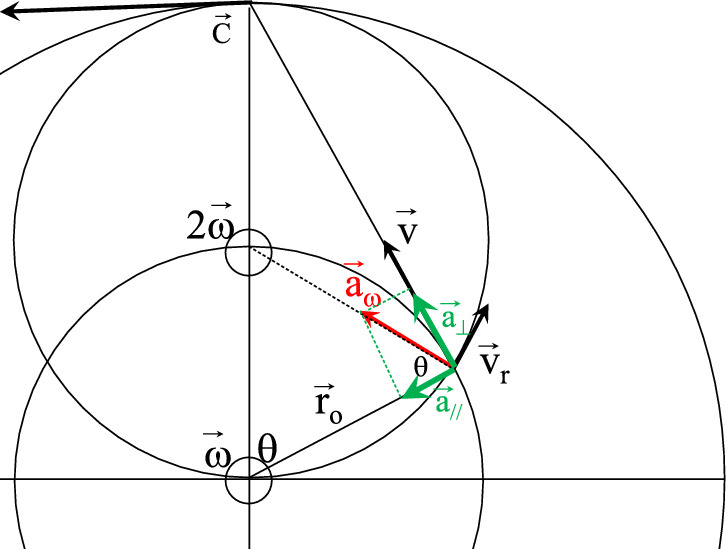
Relativistic rotation of a satellite around a central observer. A part of the Coriolis acceleration ${\overrightarrow{\mathrm{a}}}_{\upomega }=2\overrightarrow{\upomega }\wedge {\overrightarrow{\mathrm{v}}}_{\mathrm{r}}$ has a radial component that is added on top of the classical gravitational attraction: this is the magnetic effect described by Josef Lense and Hans Thirring.

Let us now turn our attention toward a mechanistic approach through relativistic rigid body rotation. By construction of the rigid body rotation, the physical or real radial axis for a central observer is the curvilinear abscissa along the space circle. As a consequence, the orthoradial axis is parallel to a radius of the space circle. For the central observer, the velocity vector direction is a tangent of the velocity circle and thus presents a non-zero radial component v_r_, Fig. 2. Thus, the acceleration formulated by Gustave Coriolis appears. This precisely applies to the radial component of the velocity which results from the relativistic distortion angle of the velocity direction. For the central observer, this acceleration can be expressed as:${\overrightarrow{\mathrm{a}}}_{\mathrm{Coriolis}}=2\overrightarrow{\upomega }\wedge \overrightarrow{{\mathrm{v}}_{\mathrm{r}}}$. It is worth noting that v_r_ = c for a photon emitted from the center. The Coriolis acceleration bends its trajectory in a way that perfectly follows a line along a circle centered on O’ with an angular speed $\overrightarrow{\mathrm{\omega {^{\prime}}}}=2\overrightarrow{\upomega }$ : this is the space circle of Eq. (3), Fig. 1a. Using relativistic transverse Doppler shift, the space circle is described since 1963^1^. It was later studied through a spatial metrical tensor borrowed to general relativity^2^. So the magnetic field reappears through a second-order term from Coriolis acceleration. Consequently, if particles captured by this magnetic field behave like satellites in circular motion, the indirect link with the original rigid body rotation through the Coriolis acceleration will explain the disappearance of any common center of rotation. This brings up one more obstacle toward a mechanical interpretation of the magnetic field. The Euler acceleration is generally stated as:

$$ {{\vec{\text a} = }}\frac{{\partial {\vec{\text{v}}}}}{{\partial {\text{t}}}}{{ + \vec{\omega }}} \wedge \overrightarrow {{{\text{v}}_{{{\varphi }}} }} {{ + 2\vec{\omega }}} \wedge \overrightarrow {{{\text{v}}_{{\text{r}}} }} . $$

Under its stationary form, the fundamental equation of GEMQ rotation is obtained and alone replaces Eqs. (1)–(3):

8 $$ {{\vec{\text a} = \vec{\omega }}} \wedge \overrightarrow {{{\text{v}}_{{{\varphi }}} }} {{ + 2\vec{\omega }}} \wedge \overrightarrow {{{\text{v}}_{{\text{r}}} }} . $$

Amazingly, although issued from classical mechanics, the relevance of the Euler equation is striking. It perfectly describes acceleration within the curved space of relativistic rotation, as illustrated in Fig. 2. The classical Euler equation becomes relativistic by abandoning the radial direction of the laboratory ${\overrightarrow{\mathrm{r}}}_{0}$ and by defining the direction induced by the Coriolis acceleration ${\overrightarrow{\mathrm{u}}}_{\mathrm{r}}=\frac{{\overrightarrow{\mathrm{v}}}_{\mathrm{r}}}{{\mathrm{v}}_{\mathrm{r}}}$, as a new radial direction of physical space which precisely defines the space circle of Eq. (3). The mathematic basis of the present theory will probably not demand the use of new tools of any exotic space nor tensor developments of general relativity but rather novel interpretations of classical mechanics in a Euclidian world.

The previous equation can be written differently, as:

9 $$ {\vec{\text{a}}} = \frac{{\partial {\vec{\text{v}}}}}{{\partial {\text{t}}}} + {\vec{\omega }} \wedge \left( {\overrightarrow {{{\text{v}}_{{{\varphi }}} }} + \overrightarrow {{{\text{v}}_{{\text{r}}} }} } \right) + {\vec{\omega }} \wedge \overrightarrow {{{\text{v}}_{{\text{r}}} }} = \frac{{\partial {\vec{\text{v}}}}}{{\partial {\text{t}}}} + {\vec{\omega }} \wedge {\vec{\text{v}}} + {\vec{\omega }} \wedge \overrightarrow {{{\text{v}}_{{\text{r}}} }} . $$

Finally, if the gradient term related to the electric field is maintained by ignoring its true curl nature, the following equation is obtained:

$$ {\vec{\text{a}}} = \frac{{\partial {\vec{\text{v}}}}}{{\partial {\text{t}}}} - \overrightarrow {{{\text{grad}}}} \frac{{{\text{v}}^{2} }}{2} + {\vec{\omega }} \wedge \overrightarrow {{{\text{v}}_{{\text{r}}} }} . $$

The projection of Coriolis acceleration in radial and orthoradial directions of the laboratory frame gives:

$$ \left\{ {\begin{array}{*{20}c} {\overrightarrow {{{\text{a}}_{\parallel }^{{\text{c}}} }} = {\vec{\omega }} \wedge {\vec{\text{v}}} {\text{cos}}^{2} \theta } \\ { \overrightarrow {{{\text{a}}_{ \bot }^{{\text{c}}} }} = \omega {\vec{\text{v}}} {\text{cos}}\theta\,\, {\text{sin}}\theta } \\ \end{array} } \right.. $$

It is worth mentioning here that these magnetic effects due to Coriolis acceleration add on top of the gravitational attraction between masses or electric attraction for charges. However, neither radial nor orthoradial acceleration implies a change in the absolute value of the velocity. They express the angular change θ that operates between the moving frame and the observer, and sustain a uniform circular motion.

The relativistic Euler equation that was expected is thus:

10 $$ {\vec{\text{a}}} = \frac{{\partial {\vec{\text{v}}}}}{{\partial {\text{t}}}} - \overrightarrow {{{\text{grad}}}} \frac{{{\text{v}}^{2} }}{2} + {\vec{\omega }} \wedge {{\vec{v} {\text {cos}}}}^{2} {\uptheta } + {{\omega \vec{v} {\text {cos}}\theta {\text {sin}}\theta }}{.} $$

If the transient regime term $\frac{\partial \overrightarrow{\mathrm{v}}}{\partial \mathrm{t}}$ is removed, the expression for a stationary regime is:

11 $$ {\vec{\text{a}}} = - \overrightarrow {{{\text{grad}}}} \frac{{{\text{v}}^{2} }}{2} + {\vec{\omega }} \wedge {{\vec{v} {\text {cos}}}}^{2} {\uptheta } + {{\omega \vec{v} {\text {cos}}\theta\,\, {\text {sin}}\theta }}{.} $$

The aim is to deduce equations of electromagnetism from this latter Euler acceleration within a relativistic rotation. On top of geometric considerations, a physical system, such as an electron caught in an electromagnetic field is needed. Expressions of mass, of charge and of electromagnetic field are missing here. Nevertheless, a qualitative comparison with a result of literature obtained from relativistic equations of electromagnetic field^3^ confirms the relevance of the Eulerian approach of Maxwell equations.

12 $$ {\vec{\text{a}}} = \frac{{\text{e}}}{{\text{m}}}\sqrt {1 - \frac{{{\text{v}}^{2} }}{{{\text{c}}^{2} }}} \left\{ {{\vec{\text{E}}} + \frac{1}{{\text{c}}}{\vec{\text{v}}} \times {\vec{\text{H}}} - \frac{1}{{{\text{c}}^{2} }}{\vec{\text{v}}}\left( {{\vec{\text{v}}} \cdot {\vec{\text{E}}}} \right)} \right\}. $$

A term to term identification of Eqs. (11) and (12) shows the electric field as a gradient of energy as expected in the beginning of this section. Second, the presence of magnetic force results from a rigid body rotation. The case of a moving charge at the origin of magnetic field is well known as Faraday’s induction. A neutral mass in rotation inducing a rotation field is way less famous but has however been described in the frame of General Relativity and is known as the Lense-Thirring effect. This was recently demonstrated in experiment^4^. This second order force with respect to velocity naturally appears in the theory as a direct consequence of the topologic angular distortion experienced by the referential frame in circular motion. Its origin is more precisely, the Coriolis acceleration, just as the last term of the equation. This last term, is a first order term and with a component parallel to the satellite velocity, plays a central role during transient phase of acceleration, deceleration and probably more generally, during energy exchange with other systems.

From relativistic rigid body rotation to relativity.

Except for the natural introduction of a maximum speed and the Lense-Thirring effect, the relativistic rigid body rotation introduced in Fig. 1 does not quite deserve its first adjective “relativistic”. This section is specifically devoted to geometric interpretation of the relativistic rigid body rotation within the landscape of the space and time perceptions of special relativity.

**Figure 3:**
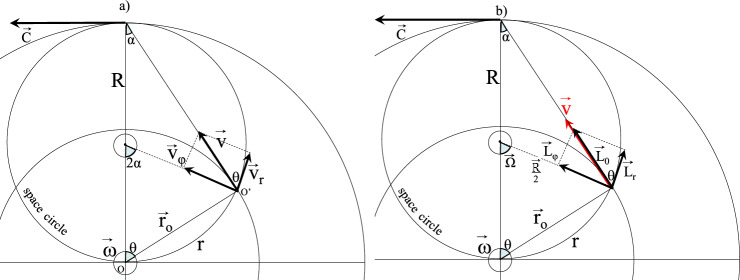
Relativistic rigid body rotation of a satellite. (**a**) The rotation velocity $\overrightarrow{\mathrm{v}}$ appears from the central observer O in referential ℜ with a radial components v_r_. (**b**) A proper length ${\mathrm{L}}_{0}$ appears from O through its orthoradial component ${\mathrm{L}}_{\mathrm{\varphi }}$.

In Fig. 3a, four reference frames can be defined: the first one is attached to the central point of observation O. It is the Galilean reference frame ℜ_G_, a mathematical fixed referential with horizontal x, vertical y, and out of plane z (parallel to $\overrightarrow{\upomega }$) axes. The second reference frame ℜ_0_ is also attached to O but in rotation motion at the angular speed ω. A cylindrical coordinate system is associated to ℜ_0_ with directions $\left( {{\vec{\text{u}}}_{ \bot } = \frac{{{\vec{\text{v}}}}}{{\left\| {{\vec{\text{v}}}} \right\|}},{\vec{\text{u}}}_{\parallel } = \frac{{{\vec{\text{r}}}_{0} }}{{\left\| {{\vec{\text{r}}}_{0} } \right\|}},{\vec{\text{u}}}_{{\text{z}}} = \frac{{{\vec{\omega }}}}{{\left\| {{\vec{\omega }}} \right\|}}} \right)$. This reference frame is also a purely mathematical construction and does not fully obey the laws of physics. Indeed, a light ray sent from O would not follow direction ${\overrightarrow{\mathrm{u}}}_{\parallel }$. The third reference frame ℜ is also attached to O and, is also in rotation at the angular speed ω but it differs in the directions of the associated cylindrical coordinate system $\left( {{\vec{\text{u}}}_{{{\varphi }}} = \frac{{{\vec{\text{v}}}_{{{\varphi }}} }}{{\left\| {{\vec{\text{v}}}_{{{\varphi }}} } \right\|}},{\vec{\text{u}}}_{{\text{r}}} = \frac{{{\vec{\text{v}}}_{{\text{r}}} }}{{\left\| {{\vec{\text{v}}}_{{\text{r}}} } \right\|}},{\vec{\text{u}}}_{{\text{z}}} = \frac{{{\vec{\omega }}}}{{\left\| {{\vec{\omega }}} \right\|}}} \right)$. The latter trihedrons are rotated by the angle α around direction ${\overrightarrow{\mathrm{u}}}_{\mathrm{z}}$. The special feature of this latter referential ℜ compared to the classical cylindrical system ℜ_0_ is to indicate the tangent of the space circle as radial direction. It is the space circle that for any observer in a laboratory describes the outer direction. ℜ is associated to a physical frame of reference, in the sense that the radial direction ${\overrightarrow{\mathrm{u}}}_{\mathrm{r}}$ perfectly follows the light path of a ray sent from O. The last reference frame, attached to O’ and traveling at velocity $\overrightarrow{\mathrm{v}}$ is the local satellite referential ℜ’. Observed from ℜ, the local metric is modified in a way that the coordinate system directions are not perpendicular any more $\left( {\overrightarrow {{{{{\text u}^{\prime}}}}} _{{\text{y}}}  = \frac{{{{\vec{v}}}}}{{\left\| {{{\vec{v}}}} \right\|}},\overrightarrow {{{{{\text u}'}}}} _{{\text{x}}}  = \frac{{{{\vec{v}}}_{{\text{r}}} }}{{\left\| {{{\vec{v}}}_{{\text{r}}} } \right\|}},{{\vec{u}}}_{{\text{z}}}  = \frac{{{{\vec{\omega }}}}}{{\left\| {{{\vec{\omega }}}} \right\|}}} \right)$. The angular distortion measured from O, $(\widehat{{\overrightarrow{\mathrm{u{^{\prime}}}}}_{\mathrm{y}},{\overrightarrow{\mathrm{u{^{\prime}}}}}_{\mathrm{x}}})=\uptheta$ explains the origin of a radial component v_r_ of the velocity field $\overrightarrow{\mathrm{v}}$. It can be recalled (section “Introduction”) that construction of the cosine of this angle takes the form:

13 $$ {{{\text cos}\theta }} = \frac{{{\text{r}}_{0} }}{{\text{R}}} = \frac{{\text{v}}}{{\text{c}}}. $$

Observed from ℜ, the velocity $\overrightarrow{\mathrm{v}}$ is a tangent to the orbit circle and not parallel to ${\overrightarrow{\mathrm{u}}}_{\mathrm{\varphi }}$ anymore; it acquires a radial component v_r_. Moreover, any length L_O_ transported within the satellite and aligned with $\overrightarrow{\mathrm{v}}$, Fig. 3b is seen by its projection along ${\overrightarrow{\mathrm{u}}}_{\mathrm{\varphi }}$, and thus appears smaller by the quantity $\mathrm{sin\theta }$:

14 $$ {\text{L}}_{{{\varphi }}} = {\text{L}}_{0} \,\,{{{\text {sin}}\theta }}{.} $$

This is the contraction of length in motion along direction $\overrightarrow{\mathrm{v}}$. Length contractions are a consequence of the angular distortion between referential ℜ and ℜ′. It can be noted that radial length along the space circle is not affected by any contraction as this direction is shared by ℜ and ℜ′, ${\overrightarrow{\mathrm{u{^{\prime}}}}}_{\mathrm{x}}={\overrightarrow{\mathrm{u}}}_{\mathrm{r}}$. As a direct consequence of the length contraction caused by the angular distortion, the parallel component of a moving surface becomes $\mathrm{S}={\mathrm{S}}_{0}\mathrm{ sin\theta }$, and a moving volume becomes $\mathrm{V}={\mathrm{V}}_{0}\mathrm{ sin\theta }$.

Time dilatation of a clock attached to the referential in motion ℜ’ from an observer within the laboratory follows the same logic issued from the local metric distortion of distance. Time dilatation is not an independent phenomenon. The orthoradial component of velocity v_φ_ is decreased by $\mathrm{sin\theta }$ when compared to the rotation velocity v. Two options are possible: ${\mathrm{v}}_{\mathrm{\varphi }}=\frac{\mathrm{d}}{\mathrm{t}}$ decreases because distance d is contracted, the case examined in the previous section, or because t dilates, in other words the rigid body rotation ω slows down. ‘Dilatation’ is a sibylline terminology. It means that the time indicated by a clock linked to a motionless observer runs faster that time of a moving clock. As a consequence, for an observer placed in the center O of rigid body rotation, any clock (rigid body rotation) with an intrinsic period ${\uptau }_{0}$ or pulsation ω_0_ and moving at a velocity v is perceived with an increased period $\uptau =\frac{{\uptau }_{0}}{\mathrm{sin\theta }}$ or with a decreased pulsation:

15 $$ {\upomega } = {\upomega }_{0} {{{\text {sin}}\theta }}{.} $$

It is worthy to mention here that rotation has been shown to be compatible with special relativity^5,6^. However, an original Euclidian geometric interpretation of Lorentz transformation is proposed now.

In the special relativity theory, transformation of space and time operates between inertial referential frames according to the Lorentz transformations:

16 $$ \left\{ {\begin{array}{*{20}l} {x = \frac{{{{{\text x}^{\prime}}} + {{{\text vt}^{\prime}}}}}{{\sqrt {1 - \frac{{{\text{v}}^{2} }}{{{\text{c}}^{2} }}} }}} \\ {t = \frac{{{{{\text t}^{\prime}}} + \frac{{\text{v}}}{{{\text{c}}^{2} }}{{{\text x}^{\prime}}}}}{{\sqrt {1 - \frac{{{\text{v}}^{2} }}{{{\text{c}}^{2} }}} }}} \\ \end{array} } \right.. $$

These transformations express the invariance of the unsurpassable speed of light and were thus discovered from the Maxwell equation. Historically^7^, after having unsuccessfully applied the Galilean transformation to the Maxwell equations, Poincarré had the reverse idea to apply Lorentz transformations to laws of mechanics. The mechanical or geometrical interpretation of the Lorentz transformations remained an enigma until Einstein introduced them as a special rotation class of quadrivectors in a Minkowski space. The elegance of this mathematic formalism logically seduced the world of physicists, who adopted the Einstein point of view once and for all^8^: “* (…) the Lorentz transformation so defined is identical with the translation and rotational transformations of the Euclidian geometry, if we disregard the number of dimensions and the relations of reality*”. By including special relativity in a wider theory that Einstein called “general relativity”, Landau^9^ expressed the general feeling shared by most physicists today: “(…) this is for sure the most beautiful of the physics theories.” Nevertheless, here an alternative interpretation of the Lorentz transformations is proposed, based on Euclidian geometry and connected to the above-mentioned ‘reality’ of Einstein. The leading idea is to merge the four spatio-temporal variables (x, y, z, t) into three physical dynamic entities ($\overrightarrow{\upomega },\overrightarrow{\mathrm{ r}},\overrightarrow{\mathrm{ v}}$) that implicitly contains Time.

The Lorentz transformation (16) can be written in a more geometrical way by using angles θ and θ’ defined as $\mathrm{cos\theta }=\frac{\mathrm{v}}{\mathrm{c}}$ and $\mathrm{cos\theta {^{\prime}}}=\frac{\mathrm{v{^{\prime}}}}{\mathrm{c}}$. v is the velocity of the frame center O′ from observer O, $\mathrm{v{^{\prime}}}=\frac{\mathrm{x{^{\prime}}}}{\mathrm{t{^{\prime}}}}$ is the velocity of the satellite spinning around O′.

17 $$ \left\{ {\begin{array}{*{20}l} {x = ct^{\prime}\frac{{{{{\text {cos}}\theta^{\prime}}} + {{{\text {cos}}\theta }}}}{{{{{\text {sin}}\theta }}}}} \\ {t = t^{\prime}\frac{{1 + {{{\text {cos}}\theta {\text {cos}}\theta^{\prime}}}}}{{{{{\text {sin}}\theta }}}}} \\ \end{array} } \right.. $$

Let us begin with examination of the simple case for which cosθ′ = 0; i.e. v′ = 0 or x′ = 0. The resulting Lorentz transformations at work on the origin O′ of the coordinate system in motion R′ are:

18 $$ \left\{ {\begin{array}{*{20}l} {x = {ct^\prime} \frac{{{{{\text {cos}}\theta }}}}{{{{{\text {sin}}\theta }}}}} \\ {t = \frac{{{{{\text t}^{\prime}}}}}{{{{{\text {sin}}\theta }}}}} \\ \end{array} } \right.. $$

**Figure 4:**
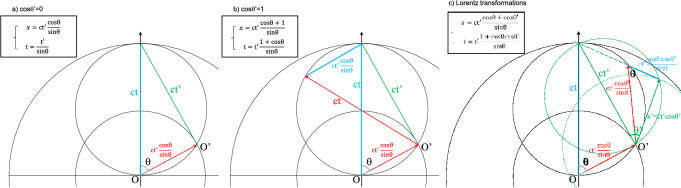
Relativistic rigid body rotation and geometrical interpretation of Lorentz transformations when cosθ′ = 0 (**a**), cosθ′ = 1 (**b**), and general case (**c**). The red color is chosen for space segments, blue for time segments as they are measured from O and green is from O′.

This special case of the Lorentz transformations is perfectly compatible with the relativistic rigid body rotation. In Fig. 4a, the distances ct, ct′, and $\mathrm{v}=\mathrm{ct cos\theta }$ are interpreted as the hypotenuse, the opposite side and the adjacent side of the rectangle triangle inscribed within the relativistic rotation circle of center O around which a satellite revolves on O′ at constant speed v. The relativistic rotation first introduced intuitively as a starting point of the theory proves to be a special case of the Lorentz transformations. As mentioned in the previous sections, the relativistic term $\frac{1}{\mathrm{sin\theta }}$ has a geometrical interpretation as an angular distortion between the velocity direction and the space circle direction. This interpretation is hidden in special relativity theory within hyperbolic functions that describe rotation of a (x, y, z, t) quadrisystem. Piloted by this angular distortion, the ratio of clock beatings from O and O′, according to the second Eq. (18), agrees perfectly with the pulsation decrease of the rotation rate of Eq. (15). In agreement with^2^, this offers the possibility for common synchronization of all points of the space circle by a simple sinusoidal term. If an isotropic pulse is sent from O, the whole clocks of the rotation are synchronized. In addition, based on the first Eq. (18), the space segment x = vt is represented in red in Fig. 4a. Similarly, based on the second part of Eq. (18) multiplied by the speed of light, the ‘time’ segment ct is represented in blue in Fig. 4a. This is the time beating of clock from O, which is proportional to the time needed for light to propagate around one horizon tour.

Let us now examine a second simple extreme case for which cosθ′ = 1. This situation corresponds to a body traveling at the speed of light v′ = c from the referential of satellite O’. In this moving referential at velocity v from O, the situation is quite simple as the time and space segments are both equal to ct′. However, from the rotation center O, the space and time segments are dissociated in two parts; the first *original* segment corresponds to the motion of O′ whereas the *additional* segment corresponds to the motion of the satellite body, Fig. 4b. The red space original segment $\mathrm{ct{^{\prime}}}\frac{\mathrm{cos\theta }}{\mathrm{sin\theta }}$ corresponds, as in the previous case, to the light traveling distance r. The red space additional segment ct represents the light traveling distance R. The blue time original segment ct corresponds, as in the previous case, to the propagation time of light along the distance R. The blue time additional segment, $\mathrm{ct{^{\prime}}}\frac{\mathrm{cos\theta }}{\mathrm{sin\theta }}$ is the propagation time of light along the distance r and therefore can be interpreted as a temporal perspective that increases with distance. Responsible for the disappearance of synchronism between moving frames^10^, this latter term was the radical novelty brought by special relativity. The resulting symmetry of the red and blue pairs of segments as parts of the same rectangle in Fig. 4b, shows that the sum of the space segments is equal to the sum of the time segments, and that as a consequence, the ratio of distance to time is the speed of light c from the observer located on O. This is the invariance property of the speed of light. This symmetry of space and time, x = ct, appears immediately within the Lorentz transformations when $\mathrm{c}=\frac{\mathrm{x{^{\prime}}}}{\mathrm{t{^{\prime}}}}$ is used in Eq. (16) or equivalently cosθ′ = 1 in (17):

19 $$ \left\{ {\begin{array}{*{20}l} {x = ct^{\prime}\frac{{1 + {{{\rm cos}\theta }}}}{{{{{\text {sin}}\theta }}}}} \\ {t = t^{\prime}\frac{{1 + {{{\text {cos}}\theta }}}}{{{{{\text {sin}}\theta }}}}} \\ \end{array} } \right.. $$

In the general case of the Lorentz transformations, a complete representation is shown on Fig. 4c. It gets more complex but each term of Eq. (17) is faithfully represented by the time (blue) and space segments (red). This double pairs of segments find here a quite interesting interpretation. Let us examine the rectangle-triangle with a vertex O′, a side x′ (green segment) and a space segment (red) as hypotenuse. The angle $\uptheta$ is retrieved in the opposite corner to O′, in the junction of the red and blue segment (Supplement Material, Appendix A). Whatever the velocity v’, this rectangle-triangle is a small-scale head-to-tail replica of the rectangle-triangle defined in Fig. 4a for the motion of O′. Their homothetic ratio is cosθ’. The length of the additional space segment is the distance of the moving body seen from O′, ${\mathrm{x}}^{\mathrm{^{\prime}}}=\mathrm{ct{^{\prime}}}\mathrm{cos\theta {^{\prime}}}$, but corrected by the Lorentz contraction, in other word multiplied by $\frac{1}{\mathrm{sin\theta }}$. The blue adjacent side with respect to $\uptheta$ is the additional time segment. Therefore, this homothetic rectangle triangle is crucial in the definition of space–time additional segments. When the moving body travels at the speed of light c, the configuration described in Fig. 4b is retrieved as expected. Then, the two triangles have the same size and are included in the same rectangle. The additional time segment has been called the synchronism time. This sort of temporal perspective, can have the following geometrical interpretation: it is the light time of the distance from the space circle of the observer O. In other words, the synchronism time is the time needed at the speed of light to bring a satellite observed from O’ back to the circle rotation of observer O.

The ratio of space to time results in the geometrical law of relativistic speed transformation (Supplement Material, Appendix B). The last comment concerns the invariance of the interval s^2^ = c^2^t^2^−x^2^. It is obvious from a geometrical point of view from Fig. 4a where s^2^ = c^2^t′^2^ and from Fig. 4b where s^2^ = 0. It is way more difficult on Fig. 4c. However, as a general trend, it results by construction that c^2^t^2^ $\ge$ x^2^ and thus that the interval s^2^ always have a real root. Thus, the spacetime interval of the relativistic body rotation is said to be time-like or light-like but can never be space-like.

## Conclusion

As already shown in the literature, the rigid body rotation is compatible with special relativity. The novelty introduced in this manuscript is a Euclidian trigonometric construction of this relativistic rigid body rotation together with its acceleration field as observed from the central inertial frame. This geometric construction fully explains the origin of Lorentz transformation as a simple angular distortion between frames. In contrast with relativity, the postulate underlying this geometric representation is not light speed limit. The funding postulate states that straight line does not exist and that any trajectory is the result of rigid body rotation. Then light speed limit becomes a consequence of circles construction. With this postulate in mind, future works will aim at revisiting problems in physic.

## Supplementary Information

Supplementary Information.

## Data Availability

The datasets used and/or analysed during the current study available from the corresponding author on reasonable request.
