# Supplementary material for: A mechanistic interpretation of relativistic rigid body rotation
Source: Sci Rep. 2023 Jun 3;13:9047. doi: 10.1038/s41598-023-35897-9 (PMC10239445; doi:10.1038/s41598-023-35897-9)
Supplement: Supplementary file 1 — Supplementary Information. [file 41598_2023_35897_MOESM1_ESM.docx]

**Supplement material**

**Appendix A**: Additional space and time segments in Lorentz transformation

**Appendix B**: Relativistic velocity addition

**Appendix A**

Additional space and time segments in Lorentz transformation


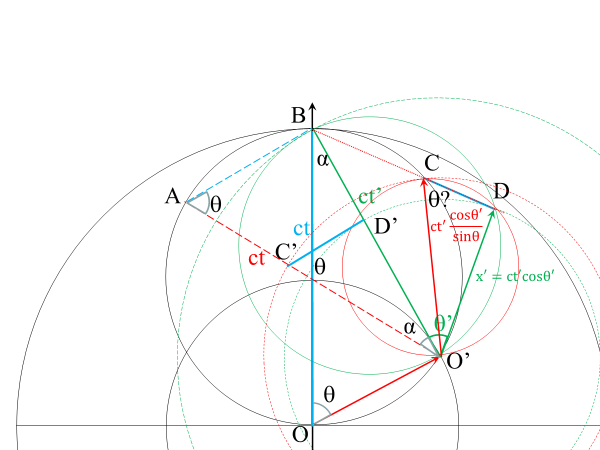


*Figure B:* *Relativistic rigid body rotation and geometrical interpretation of additional space-time segments in Lorentz transformations. A satellite body traveling at a velocity v’ is located on point D from observer O’.*

The purpose of this section is to demonstrate that the angle $\theta=\hat{O'CD}$ ; In other words, the rectangle triangle CDO’ involved in the definition of time (CD) and space (CO’) *additional* segments is a homothetic version of BOO’ involved in the definition of *original* space-time segments. Rectangle triangles ABO’ and CDO’ are confined within the black and red circles. The question of construction of CO’ as a result of the product DO’/sinθ is posed. Let us operate a rotation DO’ around O’ by the quantity θ’. D’ is obtained. In the lower part of the rectangle triangle BAO’, a homothetic rectangle triangle D’C’O’ is defined. C’O’ is equal to D’O’/sinθ and thus to DO’/sinθ, the reward length. Now it appears by construction that C’O’=CO’. More surprisingly, C becomes the intersection of the black space circle and the BD segment with the red circle of radius CO’. Therefore, rectangle triangles D’C’O’ and DCO’ have hypothenuse (red) and opposite side (green) of the same size: they are identical and result from a rotation around O’ by the angle θ’. In conclusion $\mathrm{CO}^{'}=\frac{DO'}{\sin\theta}$ and further $\theta=\hat{O'CD}$.

**Appendix B**

Relativistic velocity addition

The referential associated with the circular motion at velocity v around the central point O’ is at a distance x from an observer located on O. In this moving reference frame, a satellite located at a distance x’ from O’ has a relative velocity v’. From O, this latter segment of distance x’ is bigger by a relativistic correction factor of $\frac{1}{\sin\theta}$. Both space segments introduced up to now are represented in red. When added, they give the radius of the bigger red circle; they represent the term v+v’. As far as time segments are concerned, one has to add to the ct portion of the observer referential, the portion of the synchronism circle centered upon the opposite side of the space circle from O’. The diameter of the synchronism circle multiplied by cosθ’ exactly gives the wanted additional segments of time. When added, both time blue segments give the radius of the bigger circle; they represent the term $c(1+cos\theta\cos\theta^{'})$. The ratio of the bigger blue and red circles gives a rectangle triangle and these proportion brought back to the dimension of the rigid body rotation ω in black finally give the resultant velocity V. This component is in perfect agreement with standard relativistic speed transformation.

*Figure A:* *Two step* c*onstruction of relativistic velocity addition. Left: Lorentz transformation and space (red) and time segments constructions (blue). Right: the ratio of the sum of 2 space-segments (red) to the sum of 2 time-segments (blue) gives the final velocity vector (black). The invariance of space-time segment S is shown as a doted purple line.*
